# Modeling Systematic Change in Stopover Duration Does Not Improve Bias in Trends Estimated from Migration Counts

**DOI:** 10.1371/journal.pone.0130137

**Published:** 2015-06-18

**Authors:** Tara L. Crewe, Philip D. Taylor, Denis Lepage

**Affiliations:** 1 Department of Biology, Western University, London, Ontario, Canada; 2 Biology Department, Acadia University, Wolfville, Nova Scotia, Canada; 3 National Data Center, Bird Studies Canada, Port Rowan, Ontario, Canada; University of Missouri Kansas City, UNITED STATES

## Abstract

The use of counts of unmarked migrating animals to monitor long term population trends assumes independence of daily counts and a constant rate of detection. However, migratory stopovers often last days or weeks, violating the assumption of count independence. Further, a systematic change in stopover duration will result in a change in the probability of detecting individuals once, but also in the probability of detecting individuals on more than one sampling occasion. We tested how variation in stopover duration influenced accuracy and precision of population trends by simulating migration count data with known constant rate of population change and by allowing daily probability of survival (an index of stopover duration) to remain constant, or to vary randomly, cyclically, or increase linearly over time by various levels. Using simulated datasets with a systematic increase in stopover duration, we also tested whether any resulting bias in population trend could be reduced by modeling the underlying source of variation in detection, or by subsampling data to every three or five days to reduce the incidence of recounting. Mean bias in population trend did not differ significantly from zero when stopover duration remained constant or varied randomly over time, but bias and the detection of false trends increased significantly with a systematic increase in stopover duration. Importantly, an increase in stopover duration over time resulted in a compounding effect on counts due to the increased probability of detection and of recounting on subsequent sampling occasions. Under this scenario, bias in population trend could not be modeled using a covariate for stopover duration alone. Rather, to improve inference drawn about long term population change using counts of unmarked migrants, analyses must include a covariate for stopover duration, as well as incorporate sampling modifications (e.g., subsampling) to reduce the probability that individuals will be detected on more than one occasion.

## Introduction

Daily counts of unmarked animals migrating past or stopped at a specific geographic location (migration counts) have been used as an index of abundance to monitor long term population change, particularly for taxa that breed or winter in inaccessible, unpopulated, or otherwise unmonitored geographic regions (e.g., whales [1], songbirds [2,3], raptors [4], shorebirds [5], insects [6]). The use of daily migration counts to estimate long-term population trends relies on several assumptions, including that a new cohort of individuals is detected each day, and that the proportion of the monitored population detected remains constant over time (assumptions of count independence and proportionality, respectively)[1,7]. The assumption of count independence is likely reasonable for populations counted while actively migrating past a count site, but is more likely to be violated for populations that are counted while on migratory stopover, which for songbirds and shorebirds, can last several days or even weeks [8–10]. Regardless, violation of the assumptions of independence and proportionality are often ignored in analyses of population trends using migration counts [11,12], because, it is argued, if stopover duration and its influence on detection vary randomly and not systematically over time, annual indices of population size should provide an unbiased index of population trend.

Many factors can contribute variability to the proportion of a population detected by migration counts each day and year. Annual variation in migration route [13,14] will influence the proportion of the monitored population present to be detected at a site each year. Stopover behaviour, including daily probabilities of arrival (immigration), departure (emigration) and “survival” (1—probability of departure), and therefore stopover duration [8,15], can also vary with climate [9], weather [15,16], physiological condition [17], habitat [18,19], and presence of predators [10]. Further, individuals present at a site might be unavailable to be perceived by an observer if they are not visible or vocalizing during the sampling period [20–22]. In his review of the factors influencing the availability and perceptibility of birds, Johnson [23] suggests season, weather, observer skill, sampling effort and habitat structure are potentially confounding variables.

Unexplained variability in the various components of detection can reduce precision of monitoring programs [20], but a systematic change in detection can violate the assumption of proportionality and bias trends, leading to false inference from the data [10,22,24]. Counts of unmarked migrants reflect the proportion of the population detected by the sampling protocol [7], and detection probability is not directly estimable from the data. As a result, correlates of detection, including observer skill [25], date or local weather conditions [12,19], are often used as covariates in trend analyses to account for their potential influence on the proportion of the migrating population detected during a count. Importantly, a systematic change in stopover duration [9,10,24] in particular has the potential to influence the proportion of migrants that are detected not only once, but also the proportion of migrants that are detected on more than onecount occasion, thus violating both the assumptions of proportionality and count independence. A systematic increase in stopover duration of the Eurasian reed warbler (*Acrocephalus scirpaceus*), for example, was detected at a ringing station in Germany, and while the cause is unknown, habitat succession at that site may have played a role [24]. Alternatively, stopover duration of Western sandpipers (*Calidris mauri*) at Sydney Island, British Columbia, Canada, declined consistently from 8.4 days in 1992 to 2.7 days in 2001 in response to increasing predation risk [10]. Early migrating warblers captured at a migration monitoring site in Nova Scotia, Canada, also experienced an overall decline in stopover duration between 1996 and 2007, though inter-annual fluctuations were attributed partially to the influence of climatic cycles on stopover behaviour [9]. The effectiveness of using a covariate for detection to improve inference drawn from trends in counts of unmarked migrants when a temporal change in stopover duration has occurred has not been fully explored.

Using simulated migration count data with known constant rate of change, we tested whether systematic variation in stopover duration influenced the accuracy and precision of estimated trends in unmarked migration counts. We simulated data with previously observed low to high daily probabilities of departure [9], and therefore stopover duration, that either remained constant or varied randomly over a 20 year period. We compared results across simulated datasets with cyclic variation (e.g. in response to climatic cycles like NOA and ENSO [9]) or a systematic increase in stopover duration over the same time period. Further, we tested whether any bias in population trend that resulted from a systematic increase in stopover duration could be modeled by incorporating a covariate for annual stopover duration (assuming an independent estimate was available) or by sampling less frequently to reduce the probability of counting the same individual on more than one sampling occasion.

Conservation efforts often rely on broad-scale monitoring programs to provide assessments of population status and trend to guide management efforts (e.g., [26]). In order to use unmarked migration counts as a reliable index of population trend, it is important to understand how systematic changes in detection, and in this case stopover duration, influence trend estimates, so that appropriate measures can be taken to model, or provide caution about, these sources of error. Although our simulation is modelled on the biology of a nocturnally migrating songbird, the results are applicable to any commonly-detected species counted on migratory stopover, where counts represent the total number of individuals detected at a count site each day during a migration season over multiple years, and where individuals are unmarked and not individually identifiable (e.g. monarch butterflies, *Danaus plexippus* [6], shorebirds [5]).

## Methods

### Migration Count Simulation

Counts of unmarked migrating individuals are typically collected daily during a migration over a series of years. Sampling methods include visual counts of individuals actively migrating past a count site (e.g., raptors [11], whales [1]), census or transect counts of individuals on migratory stopover (e.g., monarchs [6], bees [27], shorebirds [5]), or as an ‘estimated total’ derived from a combination of census, visual counts, and daily banding totals from mist-netting, also on migratory stopover (e.g., songbirds [3,12,28]). We simulated migration count data for white-throated sparrows (*Zonotrichia albicollis*) counted on migratory stopover using program R [29] (See S1 Appendix and S1 Table for simulation and parameterization). White-throated sparrow counts are representative of any species that is commonly detected by migration counts, i.e., individuals are detected consistently throughout a migration with a low proportion of zero-observation days (S2 Table). The simulation model included two sub-models: an annual (among-year) model to model variability and trend in the size of the migratory count population across years, and for each year, a seasonal sub-model to model the distribution of counts among days in a year.

For the annual model, the total number of individuals migrating and available to be counted in year (*i*) one, *n*_*i = 1*_, was defined such that simulated data approximated the observed mean total count (across years) observed for the species in spring at the tip station of the Long Point Bird Observatory (LPBO), Ontario, Canada, between 1961–2011 (S2 Table). Daily estimated total data from LPBO were accessed online [30], and were collected at that site with the permission of the Ontario Ministry of Natural Resources and Forestry (OMNRF), Bird Studies Canada, and Long Point Bird Observatory, with additional permitting provided by Environment Canada—Canadian Wildlife Service (Permit Number: 10169). All of LPBO field and sampling procedures were approved by the OMNRF Animal Care and Use Committee (Protocol Number: 07–36).

Given the defined size of the count population in the first year, the total number of individuals available to be counted in all subsequent years, *n*_i>1_, was then a function of *n*_i = 1_, a defined constant rate of change (trend), *β*, random normal error on the log scale (i.e., stochastic variation in annual counts), and Poisson error on the response scale (i.e., process variation in annual counts):

$$n_{i}=Pois\left(\lambda_{i}\right)=Pois\left(\left(n_{i-1}\times \left(1+\beta \right)\right)+\varepsilon_{i}\right),\varepsilon_{i}\sim N\left(0,\sigma^{2}\right).$$

For the seasonal sub-model, *n*_*i*_ was distributed across days, *j*, in a migration season each year using a Jolly-Seber (JS) simulation model [31], which required specification of annual population size (*n*_*i*_), daily probability of arrival into the count site (*b*_*ij*_), daily probability of survival (*phi*_*ij*_) and daily probability of capture (*p*_*ij*_). Daily probability of arrival *b*_*ij*_ was modeled by first simulating a Poisson mean ‘count’ as the product of annual population size (*n*_*i*_) and a seasonal probability of movement, *s*, with temporal autocorrelation among days:

$$n_{ij}\sim Pois\left(\lambda_{ij}\right)\sim Pois\left(n_{i}\times s_{ij}\times \text{exp}\left(c\times \left(x_{ij}-\frac{j-1}{ndays_{i}-1}\right)\times npred_{i\left(j=ndays_{i}\right)}\right)\right),$$

Where *c* was a constant, *ndays* was the number of days in the migration season in year *i*, and *npred* was a function of an autocorrelation coefficient, *a*, and the previous day’s count, i.e.,

$$npred_{i\left(j+1\right)}=a\times x_{ij},$$

*x*_*ij*_ was derived from the addition of random normal error on *npred*, i.e.,

$$x_{ij}\sim N\left(npred_{ij},\sigma^{2}\right),$$

and *s*_*ij*_ was the product of a normal density and binomial probability of moving, which allowed probability of arrival at the count site to be highest mid-season during peak migration:

$$s_{ij}\sim binom\left(ndays_{i},Pm\right)\times \left({\left(2\times pi\times sigma\right)}^{-0.5}\times \text{exp}\left(-0.5\times {\left(j-\mu \right)}^{\frac{2}{sigma}}\right)\right),$$

where *Pm* was the probability of migrating on a given day, and remained constant at 0.85. The resulting *n*_*ij*_ were transformed into a daily probability of arrival, *b*_*ij*_, by scaling values to add to one.

In addition to daily probability of arrival, the JS simulation also required specification of daily probabilities of survival (*ph*_*ij*_) and capture (*p*_*ij*_). Daily probability of survival represented the binomial probability that birds ‘survived’ and remained at the count site until the following day, and is therefore directly related to daily probability of departure from the count site (1-*phi*_*ij*_), and to stopover duration [8]. We thus simulated variability in stopover duration among years by allowing survival to be constant across days in a year (i.e., *phi*_*ij*_ = *phi*_*i*_ for all days *j*) and to either remain constant (*phi*_*i*_ = 0, 0.2, 0.5, or 0.7), or to vary 1) randomly, 2) cyclically (5 year cycle), or 3) linearly among years between 0.4–0.5, 0.35–0.55, 0.3–0.6, 0.25–0.65 or 0.2–0.7 (S1 Fig). The range in probability of survival between 0.2–0.7 simulated here corresponds to the approximate range in mean departure probability observed for warblers at a migratory stopover site in Atlantic Canada [9], and a linear increase from 0.2 to 0.7 over a 20 year period resulted in a range of mean stopover duration from 1.2 to 3.4 days in our simulated datasets (S2 Fig). A constant survival probability of zero (departure probability = 1) was used as a control to simulate all birds departing after the current day’s count, which ensured independence of daily counts. Daily probability of 'capture', or in this case probability of observer detection, *p*_*ij*_, was assumed constant at 0.30 across days and years. The realized count on a given year and day (*y*_*ij*_) was the product of the sum of newly arriving individuals and individuals that survived and remained on site following the previous day’s count, and the binomial probability of observer detection, given presence. For each level stopover duration (as indexed by *phi*_*i*_) examined, we simulated 100 datasets for each of three rates of population change: a decline of 20% in 20 years (-1.2%year^-1^), no change (0%year^-1^), and an increase of 20% in 20 years (+0.96%year^-1^).

Simulation parameter values (S1 Appendix, S1 Table) were chosen such that simulated datasets approximated the distribution of real migration count data collected for white-throated sparrow at the tip station of LPBO, Ontario, Canada (1961–2011), in terms of mean and coefficient of variation (CV) of annual and daily counts, proportion of 0-observation days, and length of the migration season (S3–S6 Tables). The distribution of real and simulated count data were compared using quantile-quantile (Q-Q) plots. Correspondence of simulated and real datasets was assessed by visual inspection of Q-Q plots and by testing the Pearson correlation of Q-Q scores. A correlation coefficient near one suggests quantiles of the two datasets originate from a similar distribution of counts, even if one dataset has a higher mean count than the other (S7 Table).

### Statistical Analysis

Trend in migration counts was estimated for each simulated dataset in a Bayesian framework using integrated nested Laplace approximation using the R package R-INLA [32] in R version 3.0.0 [29] (S2 Appendix). Counts on day *j*, year *i*(*y*_*ij*_) were assumed to result from a negative binomial distribution, where *y*_*ij*_~*NegBinom*(*μ*_*ij*_,*ϕ*), where *ϕ* is the dispersion parameter. Mean counts, *μ*_*ij*_, were then fit using a log-linear regression model with year as a continuous explanatory variable to estimate population trend, and first and second order polynomial terms for day to account for the seasonal pattern of migration:

$$\text{log}(\mu_{ij})=\beta_{1}\times year_{i}+\beta_{2}\times day_{j}+\beta_{3}\times day_{j}{}^{2}+\varepsilon_{i}+\eta_{ij},$$

where *ε*_*i*_ was a random effect for year which assumed independent and identically distributed errors, i.e., *ε*_*i*_~*Normal*(0,*σ* ^2^), and *η*_*ij*_ a random effect that assumed first order autoregressive (AR1) temporal correlation of errors among days *j* in year *i* [33]. An AR1 model for the random year effect resulted in low autocorrelation (Rho) estimates, and was deemed unnecessary for these simulations. The estimated year coefficient (*β*_*1*_) was back-transformed into a trend or constant rate of population change (%year^-1^) using 100×(exp(*estimate*)-1). Bias in estimated trend was then the difference between estimated and simulated trends.

For simulations with constant stopover duration, we tested whether bias in estimated trend varied among simulated factor levels by fitting a linear regression model that assumed bias of the estimated trend was a function of two factors: 1) direction of simulated trend (declining: -1.2%year^-1^; no change: 0%year^-1^, or increasing: 0.96%year^-1^) and 2) simulated survival probability (an index of stopover duration; *phi*_*i*_ = 0, 0.2, 0.5, or 0.7). For simulations where stopover duration was allowed to vary, we fit a linear regression model which assumed bias was a function of the direction of simulated trend, and an interaction between pattern of change in stopover duration (random, cyclic, or linear) and magnitude of change in stopover duration (where *phi*_*i*_ varied between 0.4–0.5,…, or 0.2–0.7). All linear regression models were fit using the *lm* function in R [29].

We then tested whether bias in estimated trend imposed by a systematic increase in stopover duration could be modeled using a covariate for detection by running a second log-linear regression model in INLA using simulated daily survival probability (a correlate of stopover duration) as an annual covariate:

$$\text{log}(\mu_{ij})=\beta_{1}\times year_{i}+\beta_{2}\times day_{j}+\beta_{3}\times day_{j}{}^{2}+\beta_{4}\times phi_{i}+\varepsilon_{i}+\eta_{ij}$$

In our case, we used perfect information on the underlying source of bias (i.e., *phi*_*i*_) as a covariate. Using real data, stopover duration (or a correlated covariate), would need to be estimated through the collection of additional data (see Discussion). However, this exercise served to test whether, under perfect conditions when stopover duration is known, a covariate is sufficient to remove any bias in estimated trend that results from a systematic change in stopover duration, given that such a change will influence both the probability of detecting an individual and the probability of recounting individuals on one or more sampling occasions. Regressions were run on datasets simulated to have a systematic increase in stopover duration and a declining trend in counts (- 1.2%year^-1^) over the 20-year period. Because any bias in estimated trend would result at least partially from an increased probability of counting the same individuals on successive days, we ran the regression on the full simulated dataset, as well as on the same datasets subset to every third or fifth observation day, to test whether subsampling can reduce bias in estimated trend by lowering the probability that an individual will be detected on more than one count occasion. Using the estimated trends, we then tested whether the addition of a covariate and/or subsampling influenced bias in trend by fitting a linear regression model (lm, R version 3.0.0 [29]) which assumed that bias was a function of a three-way interaction between magnitude of change in stopover duration (where *phi*_*i*_ increased linearly between 0.4–0.5,…, or 0.2–0.7), whether a covariate for detection probability was included or not, and whether data were subset (no subset, every three days, or every five days).

In addition to bias, we also examined how simulated variation in stopover duration, simulated rate of population change, the use of a covariate for detection and subsampling influenced accuracy and precision of trend estimates by examining 1) ‘coverage’ of credible intervals, or the proportion of simulations where the simulated trend fell within the 95% credible interval of the estimated trend, 2) ‘power’ to detect a ‘significant’ trend, or the proportion of simulations with good coverage and credible intervals that did not include zero, and 3) rate of ‘error’, or the proportion of simulations with poor coverage (simulated trend fell outside the credible interval of the estimated trend) and credible intervals that did not include zero. Rate of error describes the probability that false inference will be drawn from the data.

## Results

**Fig 1:**
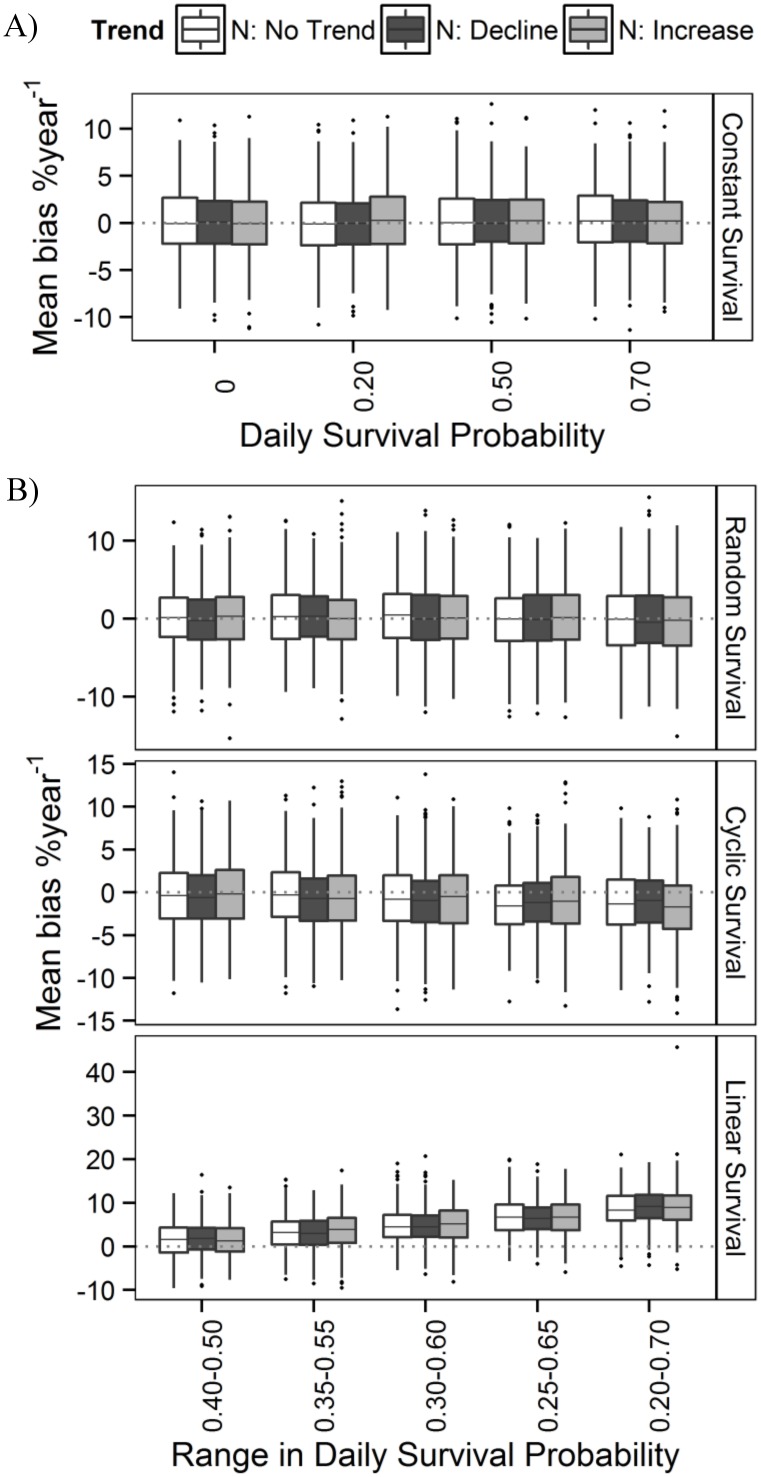
Bias in estimated population trend when stopover duration was constant or varied temporally. Bias (%year^-1^) in estimated trend in migration counts (estimated—simulated trend), when trend was estimated using datasets simulated to have either an increasing trend (0.96%year^-1^), no long term trend (0%year^-1^) or a declining trend (-1.2%year^-1^) in the count population, and where stopover duration **a)** remained constant across years (indexed by daily survival probability, *phi*_*i*_ = 0, 0.20, 0.50, or 0.70), or **b)** varied randomly, cyclically or increased systematically over time by various magnitudes (where *phi*_*i*_ varied between 0.40–0.50, 0.35–0.55, 0.30–0.60, 0.25–0.65 or 0.20–0.70). Lines of the boxplots represent the 25^th^ percentile, median and 75^th^ percentile of bias estimates across 100 simulated datasets. The horizontal dashed line depicts no bias in trend.

**Fig 2:**
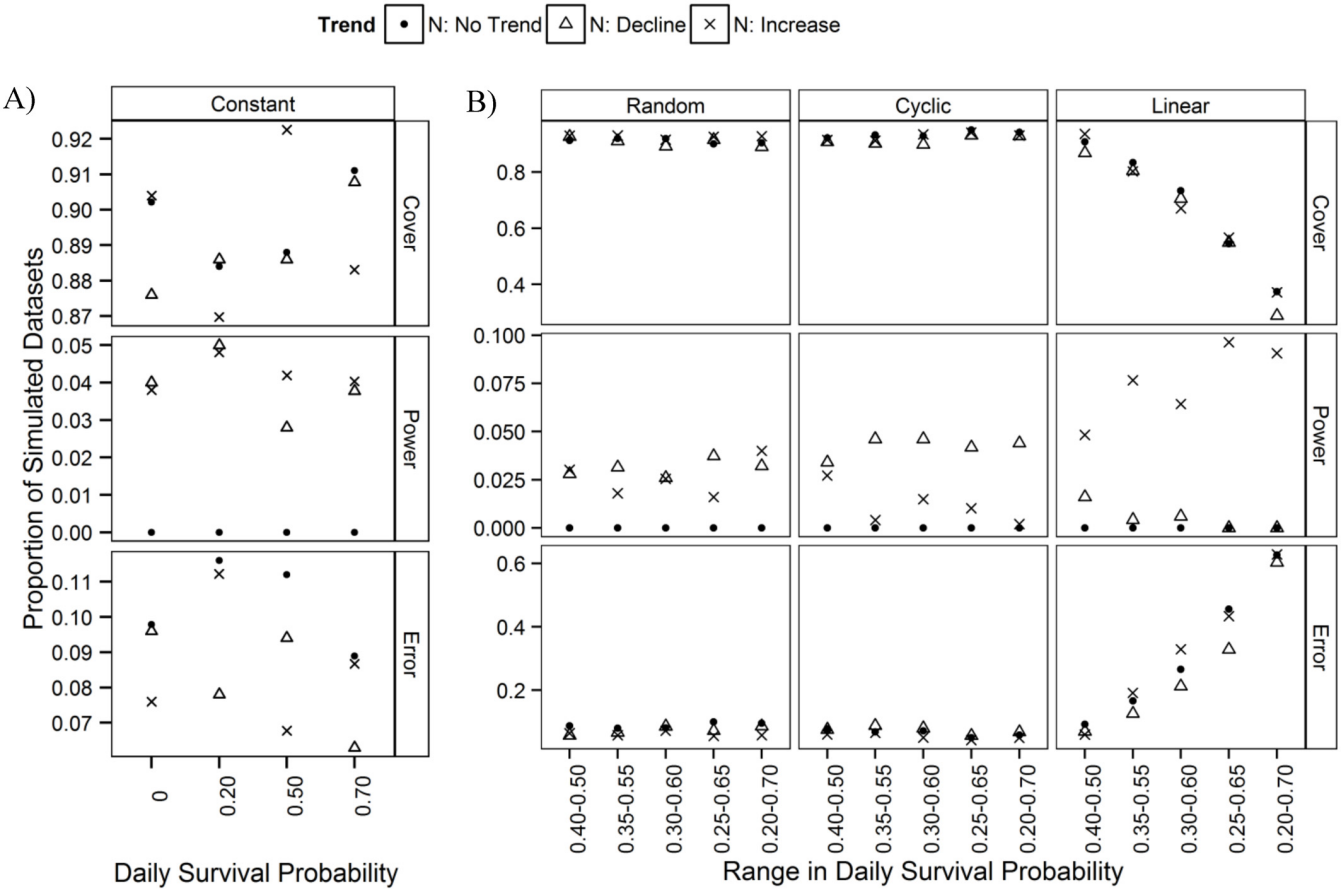
Coverage of credible intervals, power and rate of error of estimated population trends. Proportion of 100 simulated datasets with good coverage of credible intervals (simulated trend fell within credible intervals of estimated trend), power to correctly detect a ‘significant’ trend (good coverage; credible intervals did not include zero), and rate of error, or rate of falsely detecting a trend (poor coverage; credible intervals did not include zero). Results are shown for datasets simulated to have a declining trend (-1.2%year^-1^), no trend (0%year^-1^), or increasing trend (0.96%year^-1^) in the count population, and where stopover duration **a)** remained constant across years (indexed by daily survival probability, *phi*_*i*_ = 0, 0.20, 0.50 or 0.70), or **b)** varied randomly, cyclically or increased systematically among years by various magnitudes (where *phi*_*i*_ varied between 0.40–0.50 and up to 0.20–0.70).

When stopover duration was held constant, mean bias in estimated trend did not differ with direction of simulated trend (negative, no change, positive) or among simulated magnitudes of change in stopover duration (Table 1, Fig 1a). Coverage of confidence limits was greater than 86%, rate of error was less than 12%, but power was also low, at less than 6% (Fig 2a).

**Table 1:** Influence of simulated direction of trend in annual counts and length of stopover duration on bias of estimated trends, when stopover duration remained constant over time.

Parameter	Estimate	SE	t value	P
Intercept	0.00065	0.0011	0.58	0.56
Trend (-1.2%/year)	-0.00061	0.0011	-0.55	0.58
Trend (0.96%/year)	-0.00006	0.0011	-0.053	0.96
phi (0.2)	0.00012	0.0013	0.10	0.92
phi (0.5)	0.00110	0.0013	0.83	0.41
phi (0.7)	0.00169	0.0013	1.32	0.19

Reference categories for the independent variables in the linear regression model were no trend (Trend = 0%year^-1^) and no stopovers > 24 hours (*phi*_*i*_ = 0).

When stopover duration varied randomly, cyclically or increased systematically over time, mean bias in estimated trend did not differ with simulated direction of trend (Table 2, Fig 1b). However, compared to when stopover duration varied randomly, trends became increasingly positively biased as the simulated increase in stopover duration became more extreme, and to a lesser extent, increasingly negatively biased as the amplitude of cyclical change in stopover duration increased, due in part to the simulated cycle ending at a lower probability of survival than it began (S1 Fig; Table 2, Fig 1b). Credible intervals of estimated trends had greater than 85% coverage and probability of error was less than 10% when stopover duration varied randomly or cyclically (Fig 2b). However, coverage declined to less than 40% and error increased to over 60% as the magnitude of the simulated increase in stopover duration became greater (Fig 2b). Power to detect a significant trend was typically less than 5%, but for datasets simulated to have an increasing trend, an increase in stopover duration resulted in an increase in power to almost 10% (Fig 2b) due to fewer credible intervals that included zero as estimated trends became increasingly positively biased.

**Table 2:** Influence of simulated direction of trend in annual counts and magnitude and pattern of change in stopover duration (indexed by daily survival probability *phi*_*i*_) on bias of estimated trends.

Parameter	Estimate	SE	t value	P
Intercept	0.0007	0.0011	0.60	0.551
Trend(-1.2%/year)	-0.0002	0.0007	-0.31	0.759
Trend(0.96%/year)	0.0005	0.0007	0.81	0.417
Cyclic	-0.0041	0.0015	-2.83	0.005
Linear	0.0161	0.0015	11.03	<0.0001
Phi(0.35–0.55)	0.0016	0.0015	1.11	0.269
Phi(0.30–0.60)	0.0018	0.0015	1.21	0.228
Phi(0.25–0.65)	-0.0006	0.0015	-0.43	0.669
Phi(0.20–0.70)	-0.0019	0.0015	-1.31	0.192
Cyclic:Phi(0.35–0.55)	-0.0038	0.0021	-1.84	0.065
Linear:Phi(0.35–0.55)	0.0147	0.0021	7.12	<0.0001
Cyclic:Phi(0.30–0.60)	-0.0054	0.0021	-2.57	0.010
Linear:Phi(0.30–0.60)	0.0298	0.0021	14.43	<0.0001
Cyclic:Phi(0.25–0.65)	-0.0070	0.0021	-3.37	0.001
Linear:Phi(0.25–0.65)	0.0506	0.0021	24.48	<0.0001
Cyclic:Phi(0.20–0.70)	-0.0082	0.0021	-3.97	<0.0001
Linear:Phi(0.20–0.70)	0.0737	0.0021	35.54	<0.0001

Reference categories for the independent variables in the linear regression model were no trend (Trend = 0%year^-1^), random variation in stopover duration, and a range in *phi*_*i*_ values between 0.40–0.50.

**Fig 3:**
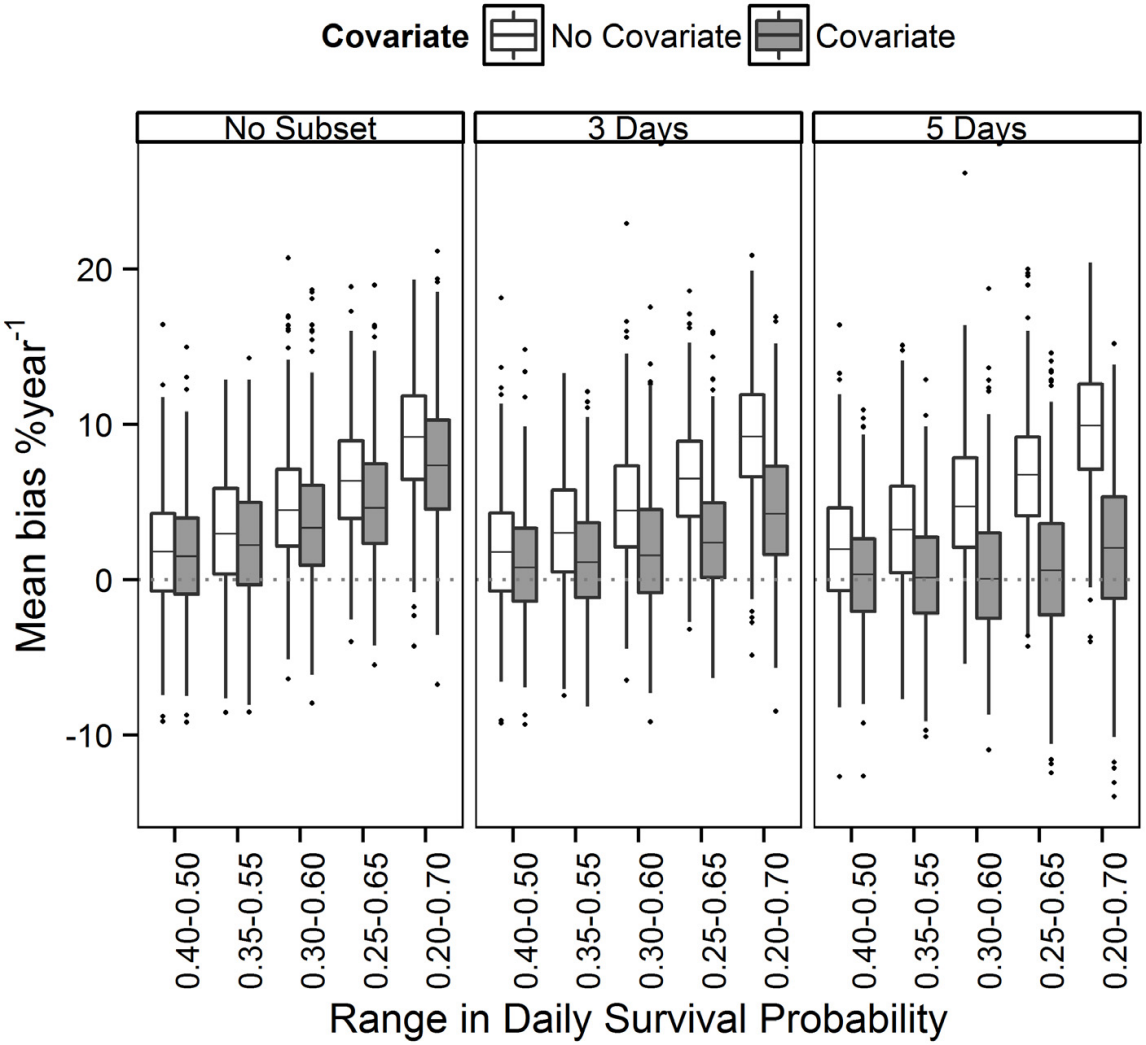
Effect of a covariate for detection and subsampling on bias of population trends. Bias (%year^-1^) in estimated trend in migration counts (estimated—simulated trend), when trend was estimated with and without a covariate for detection, and with and without subsampling to every third or fifth observation day. All datasets were simulated to have a declining population trend of 1.2%year^-1^ and a systematic increase in stopover duration (indexed by a linear increase in daily survival probability between 0.40–0.50, 0.35–0.55, 0.30–0.60, 0.25–0.65 or 0.20–0.70) over a 20-year time series. Lines of the boxplots represent the 25^th^ percentile, median and 75^th^ percentile of bias estimates across 100 simulated datasets. The horizontal dashed line depicts no bias in estimated trend.

**Fig 4:**
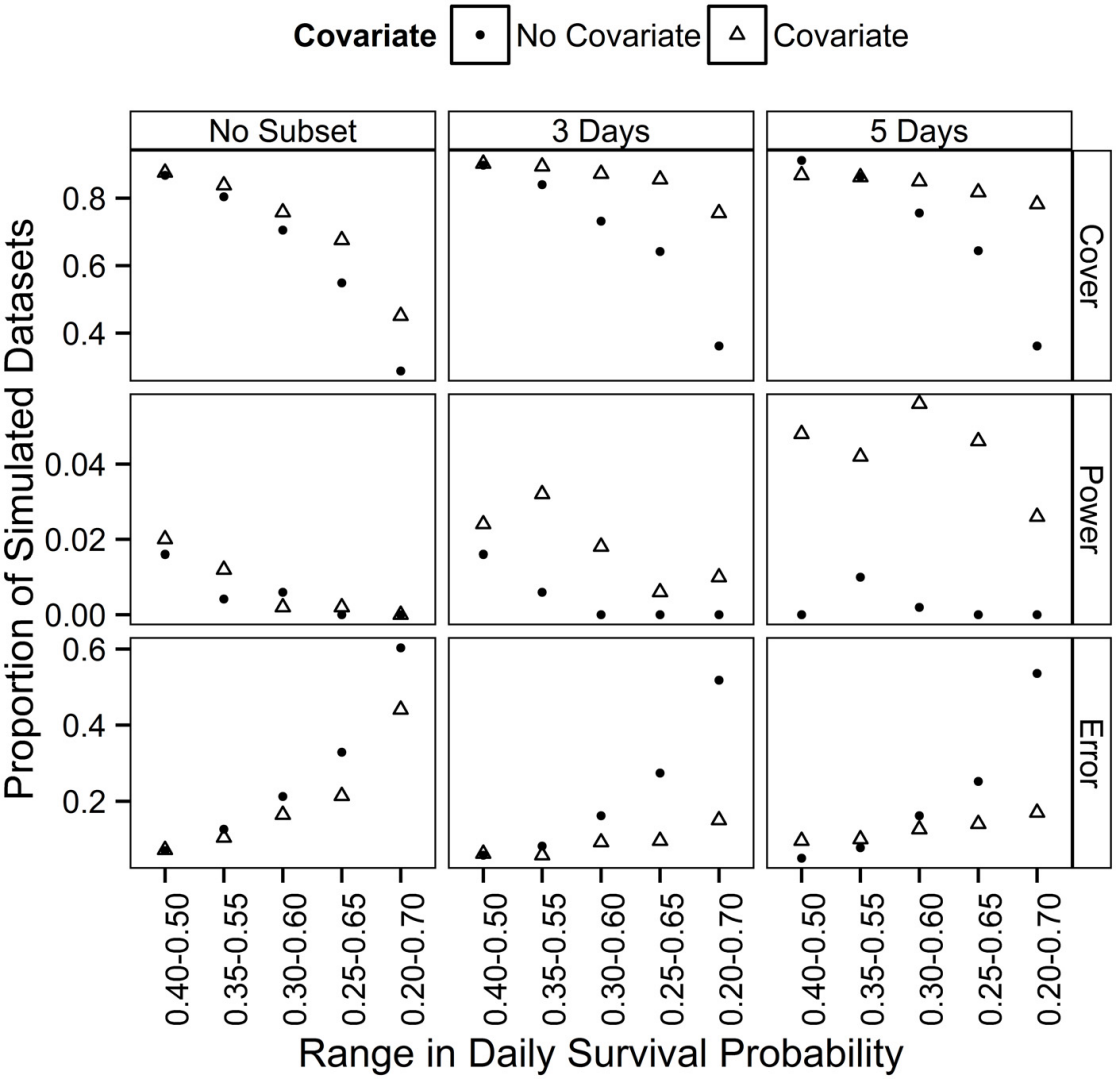
Effect of a covariate for detection and subsampling on coverage, power and rate of error of estimated trends. Proportion of 100 20-year white-throated sparrow simulated datasets with 1) good coverage of credible intervals (simulated trend fell within credible intervals of estimated trend), 2) power to correctly detect a ‘significant’ trend (good coverage; credible intervals did not include zero), and 3) error, or false detection of a trend (poor coverage; credible intervals did not include zero). Results are shown for datasets that were not subsampled or subsampled to every third or fifth observation day, and when a covariate for detection was or was not included in analysis. Datasets were simulated to have a declining trend of 1.2%year^-1^ and a systematic increase in stopover duration (indexed by a linear increase in daily survival probability between 0.40–0.50, 0.35–0.55, 0.30–0.60, 0.25–0.65 or 0.20–0.70).

Compared to datasets analyzed without a covariate or subsampling, bias in estimated trend imposed by a systematic increase in stopover duration was not influenced by subsampling data to every third or fifth observation day (Table 3, Fig 3). Trends were less biased with the inclusion of a covariate for detection, but the covariate did not remove the bias completely. Combining subsampling with a covariate for stopover duration was more successful at minimizing bias, particularly when data were subset to every fifth observation day (Fig 3). Including both a covariate and subsampling in the estimation of trends largely compensated for the effect that a systematic increase in stopover duration had on coverage of credible intervals, power and error, and for all magnitudes of change in stopover duration, resulted in over 85% coverage, less than 20% error, but power remained below 5% (Fig 4).

**Table 3:** Influence of including a covariate for detection and subsampling on bias of estimated trends, when stopover duration (indexed by daily survival probability *phi*_*i*_) increased systematically over time.

Parameter	Estimate	SE	t value	P
Intercept	0.0188	0.0018	10.57	<0.0001
Phi (0.35–0.55)	0.0122	0.0026	4.79	<0.0001
Phi (0.30–0.60)	0.0289	0.0025	11.46	<0.0001
Phi (0.25–0.65)	0.0460	0.0025	18.25	<0.0001
Phi (0.20–0.70)	0.0718	0.0025	28.22	<0.0001
Subset (3 Day)	0.0000	0.0025	-0.01	0.993
Subset (5 Day)	0.0012	0.0025	0.49	0.622
Covariate (Yes)	-0.0033	0.0025	-1.31	0.191
Phi (0.35–0.55):Subset (3 Day)	0.0007	0.0036	0.21	0.836
Phi (0.30–0.60):Subset (3 Day)	0.0006	0.0036	0.17	0.868
Phi (0.25–0.65):Subset (3 Day)	0.0009	0.0036	0.25	0.805
Phi (0.20–0.70):Subset (3 Day)	0.0013	0.0036	0.36	0.721
Phi (0.35–0.55):Subset (5 Day)	0.0006	0.0036	0.16	0.876
Phi (0.30–0.60):Subset (5 Day)	0.0012	0.0036	0.33	0.744
Phi (0.25–0.65):Subset (5 Day)	0.0024	0.0036	0.67	0.506
Phi (0.20–0.70):Subset (5 Day)	0.0061	0.0036	1.71	0.087
Phi (0.35–0.55):Covariate (Yes)	-0.0034	0.0036	-0.95	0.342
Phi (0.30–0.60):Covariate (Yes)	-0.0076	0.0036	-2.13	0.033
Phi (0.25–0.65):Covariate (Yes)	-0.0108	0.0036	-3.04	0.002
Phi (0.20–0.70):Covariate (Yes)	-0.0138	0.0036	-3.85	0.0001
Subset (3 Day):Covariate (Yes)	-0.0057	0.0036	-1.61	0.108
Subset (5 Day):Covariate (Yes)	-0.0124	0.0036	-3.47	0.001
Phi (0.35–0.55):Subset (3 Day):Covariate (Yes)	-0.0059	0.0050	-1.18	0.240
Phi (0.30–0.60):Subset (3 Day):Covariate (Yes)	-0.0121	0.0050	-2.40	0.016
Phi (0.25–0.65):Subset (3 Day):Covariate (Yes)	-0.0183	0.0050	-3.65	0.0002
Phi (0.20–0.70):Subset (3 Day):Covariate (Yes)	-0.0256	0.0050	-5.08	<0.0001
Phi (0.35–0.55):Subset (5 Day):Covariate (Yes)	-0.0102	0.0050	-2.01	0.044
Phi (0.30–0.60):Subset (5 Day):Covariate (Yes)	-0.0237	0.0050	-4.70	<0.0001
Phi (0.25–0.65):Subset (5 Day):Covariate (Yes)	-0.0346	0.0050	-6.88	<0.0001
Phi (0.20–0.70):Subset (5 Day):Covariate (Yes)	-0.0489	0.0050	-9.68	<0.0001

Reference categories for the independent variables in the linear regression model were an increase in *phi*_*i*_ between 0.40–0.50, no subset, and no covariate.

## Discussion

Conservation efforts rely on monitoring programs to guide management priorities through accurate and precise assessments of population status and long-term trend [26]. However, ecological systems are inherently complex and variable, and in the analysis of time series data, the potential exists for any number of factors to generate a systematic change in the proportion of a population that is detected [21,22]. If left unaccounted for, a systematic change in detection can lead to the estimation of false population trends [21,22,24]. Our results support the assertion that a violation of the assumption of proportionality through a systematic increase in probability of detection, and specifically in stopover duration, will bias trends and lead to a higher probability of drawing false inference from migration count data. As stopover duration increased with an associated linear increase in daily survival probability from 0.25 to 0.65 or from 0.20 to 0.70 in 20 years, probability of error, and therefore probability of drawing false inference from the data, exceeded 60%. Compared to previously observed rates of change in departure probability for early migrating warblers (0.2–0.8 in 11 years) [9] and in stopover duration for shorebirds (8.4–2.7 days in 10 years) [10], the probability of falsely detecting a trend observed here should be considered conservative. To a lesser extent, cyclical variation in stopover duration also has the potential to bias estimated trends when the amplitude of fluctuations is large and cycles aren’t completed during the time span analyzed, which in real situations cannot be known. Random variation in stopover duration did not bias trends, but as expected, the resulting unexplained variation in migration counts did result in lower power compared to when stopover duration remained constant.

In order to improve inference of population trends derived from counts of unmarked migrants, monitoring programs often use data on correlates of detection, including weather [12,19] and observer [25], as covariates in trend analyses. However, when the assumption of count independence is violated by a systematic increase in the length of migratory stopover, our results suggest that a reliance on covariates to model detection probability is not sufficient to improve inference of population trends. This is true even though the covariate we tested represented the known underlying factor influencing detection probability, without estimation error. As stopover duration increased over time, individuals became more likely to stay on site and be detected on an increasing number of sampling occasions. Lacking the ability to exclude recaptures, as is typically done using counts of marked birds on migratory stopover [19,34], the resulting inflation or compounding effect on daily counts over time was not accounted for by a covariate for detection probability alone. Rather, our results suggest that in order to minimize the probability of drawing false inference from unmarked migration count data, analyses of population trends must model the underlying change in stopover duration, as well as incorporate modifications to the sampling protocol to either exclude ‘recaptures’ (e.g. only count birds newly arriving at a site) or minimize the probability that individuals will be counted on more than one sampling occasion. These modifications would also be required if stopover duration were declining instead: in this case, individuals would become less likely to be detected on one or more sampling occasions over time, such that counts would become less inflated by recounting, and estimated population trends more negative than actual. Alternatively, while rarely detected species would be subject to similar biases, the sporadic presence of only one or a few individuals at a site at a time may make the exclusion of probable or known recaptures from daily totals possible, and would essentially limit observations to first captures as with marked migration counts [34]. In this case, a systematic change in any factor influencing detection could still bias trends [10,19,24] and should be modeled, but subsampling would no longer be required to limit recounting.

Ideally, monitoring programs would be designed to allow for the direct estimation of detection and recapture. Temporally or spatially replicated counts of territorial animals (i.e., under the assumption of population closure) collected annually, for example, can be used to estimate population trend while explicitly modeling components of detection [21,22,35]. This would likely be considered the ideal sampling protocol to monitor population abundance and distribution ofcommonly detected species with accessible breeding grounds [35,36]. However, migration monitoring typically targets species that are either secretive breeders not commonly detected by breeding surveys (e.g., raptors [11]), or species that breed in inaccessible, remote locations, where breeding surveys can be logistically or financially unrealistic [1,12]. This is the case for many long-distance landbird migrants that breed in the northern and boreal forests of Canada. A large proportion of the breeding population of many of these species lies north of human populated regions [37], and therefore beyond the range of other large-scale monitoring programs (e.g., Breeding Bird Survey [38]). Migration monitoring has been identified as an important source of data for these northern-breeding populations [26].

The use of daily capture-recapture sampling protocols to monitor migrating populations allows recaptures to be excluded [34] and variability in stopover parameters (e.g., trappability, departure probability, stopover duration) to be modeled and accounted for when estimating population trends [24]. Currently, a majority of member sites of the Canadian Migration Monitoring Network (CMMN), which focuses primarily on monitoring populations of long-distance landbird migrants, do collect banding data in addition to daily estimated totals of unmarked migrants [3]. For species that are detected in sufficient numbers, the use of banding data to estimate population trends in a mark-recapture framework that accounts for variation in detection probability [24] should be considered a preferred alternative over estimating trends using counts of unmarked animals. However, a majority of species that pass through migration count sites aren’t captured in sufficient numbers to be analyzed in a mark-recapture framework [9,24]. Indeed, this is a primary reason why Hussell and Ralph [28,39] recommend combining multiple count methods into a daily estimated total for migratory landbird monitoring. The use of two or more count methods to derive a daily estimated total allows a greater number of species to be detected in numbers sufficient for analysis, and allows counts to be estimated during poor weather conditions when nets used for banding are typically closed [28,39].

In order to improve estimates of population trend using counts of unmarked migrants, we recommend the collection of independent data on the various components of detection, to be included as covariates in population trend analyses, or as components of the underlying models themselves. For example, incorporating radar and acoustic monitoring data into estimates of daily migration volume [40,41] could be profitable. In addition, stopover parameters (including stopover duration) could be estimated for a given species or species group using local band recoveries [9,24], large-scale automated telemetry arrays [42], or other mark-recapture techniques. Further, probability of observer detection could be modeled either through the collection of independent data on sampling effort or observer skill [25], or through the use of double observer or other repeated sampling approaches [5,23,43]. Where independent data on detection are not available, correlates of detection (e.g., weather, date) should be used as covariates in trend analyses [12,19].

When individuals are known to stop at a count site for extended periods of time and recaptures cannot be excluded from daily counts, the incidence of multiple-counting should be addressed through subsampling or other modifications to the sampling protocol. Hussell and Ralph [28,39] suggest recording and subtracting the number of probable or known stopovers from daily counts of migrating landbirds, which can be calculated directly from band recoveries (where available) or estimated based on observer knowledge of the count site and individuals present. At sites where stopover duration is typically short and independent data on stopover parameters are collected, the omission of days with a low estimated probability of departure should be tested for its effectiveness in reducing both the incidence of multiple counting and bias of estimated population trends. Compared to subsampling, the latter method has the potential to minimize a reduction in sample size and therefore power. Count sites should also be placed in locations with a high turnover of migrants, such as exposed coastal sites that funnel migrants but are considered poor quality stopover habitat, to reduce the probability that extended migratory stopovers will occur [28,39]. Alternatively, modifications to analytical methods can also address a change in stopover duration. Population trends for migrating shorebirds, for example, are often calculated using an estimate of annual abundance derived from the total number of individuals observed (or estimated) from daily counts, corrected by average length of stay [5,44]. This method assumes an annual estimate of stopover duration is available, and that all individuals present are observed [44]. Thus, as opposed to an index of abundance, this method provides an estimate of the total number of individuals using a site each year, and is highly sensitive to estimated length of stay [44].

Compared to the typical definition of power, which is the probability of detecting a trend that differs significantly from zero, the estimate of power we report is more conservative because it includes only simulations where the known trend also falls within the credible intervals of the estimated trend (i.e., it excludes the false detection of a significant trend, which can't be known using real data). Overall, statistical power of our analyses was low, at approximately 10% or less to detect a 20% change in the count population over 20 years. Typical of migration counts, white-throated sparrow counts collected at Long Point, and simulated here, were highly variable both within and among years (S2 and S3–S6 Tables). A similar analysis of population trends using counts of western sandpiperson migratory stopover in British Columbia, Canada, resulted in power to detect a minimum rate of change of 3.2%year^-1^, or approximately 55% in 20 years [5], which is over double the rate of population change in our simulated data. A power of 80% to detect a 50% decline in 20 years with a significance of 0.1 was suggested as a goal for landbird population monitoring [45]. The low rate of population change simulated here highlights the impact that a systematic change in detection can have on the interpretation of time series data when populations are stable or changing at a low rate relative to the change in detection. The relative impact of a given change in detection on inference drawn about population trends will decline as rate of actual population change increases. Future work should assess the minimum rate of population change required to achieve a pre-determined level of power given the magnitude of change in detection and count variability simulated here or observed elsewhere [9,24], and should include scenarios where stopover duration declines over time, which might occur with degradation of stopover site quality [10].

While a decline in stopover duration was not examined here, it is expected to result in more negative population trends than actual, i.e., estimated trends would be less extreme than actual for populations that are increasing, and more extreme than actual for populations that are declining, resulting in a lower probability of detecting a positive trend, and an increased probability of detecting a (inflated) declining trend. The sensitivity of various rates of population change to the different magnitudes of systematic change in detection should also be explored. In general, power can be improved and probability of error will decline with the use of covariates to account for variability in counts, by increasing the length of the time series, and by combining data across migration count sites that are assumed to monitor the same population. The latter would allow site-specific variation in counts, including variation in detection, to be estimated independently from underlying population change. Because different sampling protocols will be subject to different sources of detection bias [23], the use of standardized sampling protocols across sites is recommended. In addition, the assumption of proportionality is more likely to be violated as the length of a time series increases, which further emphasizes the importance of accounting for annual and daily variability in detection through the use of covariates or other method.

All sampling methods are susceptible to various potentially interacting sources of detection bias [19,23,24]. While standardization of sampling protocols is important to minimize the probability that a systematic change in detection will occur, not all sources of variability in detection can be controlled or effectively measured. The recommendation for stable habitat structure at a migration count site [28], for example, is rarely achieved [19,34,46], and can have important implications on bird behaviour and detection probability [19,46]. Mark-recapture sampling protocols provide an ideal means to monitor migrating populations while accounting for variation in detection probability [24], but unless analyzed in a guild context, sample-size requirements exclude rare species that are often the primary focus of conservation efforts. Further, even though migration counts provide only an index of population abundance that is confounded by detection probability, correspondence between bird population trends derived from migration counts and the North American Breeding Bird Survey supports their use [3,11,12]. Thus, while we recommend the collection of additional data to model detection probability, the absence of relevant covariates for detection should not preclude the use of migration counts for population trend analyses. Rather, in the absence of additional information on detection, population trends estimated using migration counts should simply state clearly and openly whether and how variation in factors known to influence stopover behaviour and detection might influence inference drawn about long-term population change.

## Supporting Information

S1 Appendix R Code for migration count simulation. (PDF)

S2 Appendix R Code to run negative binomial models using INLA. (PDF)

S1 Fig Simulated levels and pattern of change in daily probability of survival. Levels of constant, random, systematic or cyclic variation in daily survival probability (*phi*), an index of stopover duration, tested for their influence on accuracy and precision of population trends derived from unmarked migration counts. Values shown for random variation in *phi* depict one draw from a random uniform distribution. For random, systematic and cyclic variation, *phi* varied between 0.2–0.7, 0.25–0.65, 0.3–0.6, 0.35–0.55, and 0.4–0.5.
(PDF)

S2 Fig Simulated increase in daily survival probability (1- probability of departure), and associated increase in stopover duration and detections per individual. Mean (SD) of stopover duration and number of sampling occasions during which an individual was detected across 100 datasets simulated to have a linear increase in daily survival probability from 0.2 to 0.7 over a 20-year period, constant probability of observer detection (0.3), and no underlying trend in population size (0%year^-1^).
(PDF)

S1 Table Migration count simulation parameterization. Values of simulation model parameters that varied among simulated factor levels, where: ‘trend’ specifies the rate of population trend simulated; ‘phi.type’ specifies whether daily probability of survival, and therefore stopover duration, was simulated to be constant, vary randomly or cyclically, or to increase linearly over time; ‘phi.in’ specifies the rate or range in daily survival probability simulated; ‘phi.in.1’ and ‘phi.in.2’ are the minimum and maximum values of daily survival, respectively; and ‘cycle.amp’ specifies the amplitude of cyclical change required to simulate the desired range in survival/stopover duration. See S1 Appendix for migration count simulation code in R.
(PDF)

S2 Table Summary of real white-throated sparrow (*Zonotrichia albicollis*) migration count data. Mean, median, coefficient of variation (CV), minimum and maximum of migration counts collected daily at the tip station of the Long Point Bird Observatory, Ontario, Canada, during spring migration from 1961–2011.
(PDF)

S3 Table Summary of annual abundance for simulated migration count data. Mean, median and coefficient of variation (CV) of annual counts among 100 simulated datasets for each set of factor levels. Datasets were simulated to have either a declining population trend (-1.2%/year; “Decline”), no population change (0%/year; “NoChange”) or an increasing population trend (0.96%/year; “Increase”). Survival probability remained constant or varied randomly, cyclically or increased linearly over time.
(PDF)

S4 Table Summary of daily abundance for simulated migration count data. Mean, median and coefficient of variation (CV) of daily migration counts among 100 simulated datasets for each set of factor levels. Datasets were simulated to have either a declining population trend (-1.2%/year; “Decline”), no population change (0%/year; “NoChange”) or an increasing population trend (0.96%/year; “Increase”). Survival probability remained constant or varied randomly, cyclically or increased linearly over time.
(PDF)

S5 Table Summary of zero-observation days for simulated migration count data. Mean, median and coefficient of variation (CV) of the number of 0-observation days among 100 simulated datasets for each set of factor levels. Datasets were simulated to have either a declining population trend (-1.2%/year; “Decline”), no population change (0%/year; “NoChange”) or an increasing population trend (0.96%/year; “Increase”). Survival probability remained constant or varied randomly, cyclically or increased linearly over time.
(PDF)

S6 Table Summary of migration window length for simulated migration count data. Mean, median and coefficient of variation (CV) of the number of observation days each season among 100 simulated datasets for each set of factor levels. Datasets were simulated to have either a declining population trend (-1.2%/year; “Decline”), no population change (0%/year; “NoChange”) or an increasing population trend (0.96%/year; “Increase”). Survival probability remained constant or varied randomly, cyclically or increased linearly over time.
(PDF)

S7 Table Correlation of quantile-quantile plot scores comparing real and simulated migration count data. Mean (SD) of Pearson correlation coefficients of quantile-quantile (QQ) scores among 100 simulated migration count datasets with real white-throated sparrow (*Zonotrichia albicollis*) migration count data collected during spring migration at the tip station of the Long Point Bird Observatory in Ontario, Canada (1961–2011). A correlation coefficient of 1 suggests quantiles of each dataset originate from a similar distribution of counts. Four types of daily survival were tested: 1) constant (i.e., daily probability of survival was 0, 0.20, 0.50, or 0.70 across all years); or survival varied among years between 0.20–0.70, 0.25–0.65, 0.30–0.60, 0.35–0.55 and 0.40–0.50 either 2) randomly, 3) with linear/directional change, or 4) cyclically. A daily probability of survival of zero suggests all birds departed the count site within 24 hours (i.e., birds did not stop over at a site for extended periods with potential to be recaptured on subsequent counts). The lower correlation coefficients observed for simulations with constant phi is likely the result of lower mean daily and annual counts, a higher proportion of 0-observation days, and higher variability among daily counts compared to real data and to simulations with random, linear and cyclic variation in phi.
(PDF)

## References

[1] Findlay K, Best P, Meÿer M. Migrations of humpback whales past Cape Vidal, South Africa, and an estimate of the population increase rate (1988–2002). African J Mar Sci. 2011;33: 375–392.

[2] Blancher PJ, Phoenix RD, Badzinski DS, Cadman MD, Crewe TL, Downes CM, et al Population trend status of Ontario’s forest birds. For Chron. 2009;85: 184–201.

[3] Crewe TL, McCracken JD, Taylor PD, Lepage D, Heagy AE. The Canadian Migration Monitoring Network—Réseau canadien de surveillance des migrations: Ten-Year Report on Monitoring Landbird Population Change. Can Migr Monit Netw—Réseau Can Surveill des Migr Tech Rep #1. Port Rowan, Ontario, Canada; 2008; 69. Available: http://www.bsc-eoc.org/download/CMMNReport2008.pdf

[4] Bildstein KL, Smith JP, Ruelas I. E, Veit RR. State of North America’s Birds of Prey. Nuttall Ornithological Club and American Ornithologists Union Series in Ornithology No.3. Cambridge, Massachusetts, and Washington, D.C; 2008.

[5] Drever MC, Lemon MJF, Butler RW, Millikin RL. Monitoring populations of Western Sandpipers and Pacific Dunlins during northward migration on the Fraser River Delta, British Columbia, 1991–2013. J F Ornithol. 2014;85: 10–22.

[6] Gibbs D, Walton R, Brower L, Davis AK. Monarch butterfly (Lepidoptera: Nymphalidae) migration monitoring at Chincoteague, Virginia and Cape May, New Jersey: a comparison of long-term trends. J Kansas Entomol Soc. 2006;79: 156–164.

[7] Dunn EH. Counting migrants to monitor bird populations: State of the art. USDA For Serv Gen Tech Rep PSW-GTR-191. 2005; 712–717.

[8] Schaub M, Pradel R, Jenni L, Lebreton J. Migrating birds stop over longer than usually thought: An improved capture-recapture analysis. Ecology. 2001;82: 852–859.

[9] Calvert AM, Taylor PD, Walde S. Cross-scale environmental influences on migratory stopover behaviour. Glob Chang Biol. 2009;15: 744–759.

[10] Ydenberg RC, Butler RW, Lank DB, Smith BD, Ireland J. Western sandpipers have altered migration tactics as peregrine falcon populations have recovered. Proc Biol Sci. 2004;271: 1263–1269. 15306350 10.1098/rspb.2004.2713 PMC1691718

[11] Farmer CJ, Hussell DJT, Mizrahi D. Detecting population trends in migratory birds of prey. Auk. 2007;124: 1047–1062.

[12] Francis CM, Hussell DJT. Changes in numbers of land birds counted on migration at Long Point Bird Observatory. Bird Popul. 1998;4: 37–66.

[13] Vardanis Y, Klaassen RHG, Strandberg R, Alerstam T. Individuality in bird migration: routes and timing. Biol Lett. 2011;7: 502–5. 10.1098/rsbl.2010.1180 21307045 PMC3130220

[14] Alerstam T, Hake M, Kjellén N. Temporal and spatial patterns of repeated migratory journeys by ospreys. Anim Behav. 2006;71: 555–566.

[15] Schaub M, Liechti F, Jenni L. Departure of migrating European robins, Erithacus rubecula, from a stopover site in relation to wind and rain. Anim Behav. 2004;67: 229–237.

[16] Brattström O, Kjellén N, Alerstam T, Åkesson S. Effects of wind and weather on red admiral, Vanessa atalanta, migration at a coastal site in southern Sweden. Anim Behav. 2008;76: 335–344.

[17] Schaub M, Jenni L, Bairlein F. Fuel stores, fuel accumulation, and the decision to depart from a migration stopover site. Behav Ecol. 2008;19: 657–666.

[18] Russell RW, Carpenter FL, Hixon MA, Paton DC. The impact of variation in stopover habitat quality on migrant rufous hummingbirds. Conserv Biol. 1994;8: 483–490.

[19] Ballard G, Geupel GR, Nur N, Gardali T. Long-Term Declines and Decadal Patterns in Population Trends of Songbirds in Western North America, 1979–1999. Condor. 2003;105: 737–755.

[20] Nichols JD, Thomas L, Conn PB. Inferences about landbird abundance from count data: Recent advances and future directions In: Thompson DL, Cooch EG, Conroy MJ, editors. Modeling Demographic Processes in Marked Populations. New York, NY: Springer; 2009 pp. 201–235.

[21] Kéry M, Schmidt BR. Imperfect detection and its consequences for monitoring for conservation. Community Ecol. 2008;9: 207–216.

[22] Kéry M, Dorazio RM, Soldaat L, van Strien A, Zuiderwijk A, Royle JA. Trend estimation in populations with imperfect detection. J Appl Ecol. 2009;46: 1163–1172.

[23] Johnson DH. In defense of indices: The case of bird surveys. J Wildl Manage. 2008;72: 857–868.

[24] Hochachka WM, Fiedler W. Trends in trappability and stop-over duration can confound interpretations of population trajectories from long-term migration ringing studies. J Ornithol. 2008;149: 375–391.

[25] Link WA, Sauer JR. A hierarchical analysis of population change with application to cerulean warblers. Ecology. 2002;83: 2832–2840.

[26] Rich TD, Beardmore CJ, Berlanga H, Blancher PB, Bradstreet MSW, Butcher GS, et al Partners in Flight North American landbird conservation plan. Ithaca, New York, USA; 2004 Available: http://www.partnersinflight.org/cplan.htm

[27] Robinson WS. Migrating giant honey bees (Apis dorsata) congregate annually at stopover site in Thailand. PLoS One. 2012;7: e44976 10.1371/journal.pone.0044976 23028715 PMC3446981

[28] Hussell DJT, Ralph CJ. Recommended methods for monitoring change in landbird populations by counting and capturing migrants. North Am Bird Bander. 2005;30: 6–20.

[29] R Core Team. R: A language and environment for statistical computing. Vienna, Austria: R Foundation for Statistical Computing; 2013 Available: http://www.r-project.org/ 10.3758/s13428-013-0330-5

[30] Long Point Bird Observatory. Canadian Migration Monitoring Network—Daily Estimated Total. Data Accessed from NatureCounts, a node of the Avian Knowledge Network, Bird Studies Canada. 2011. Available: http://www.naturecounts.ca/

[31] Kéry M, Schaub M. Bayesian Population Analysis using WinBUGS: A hierarchical perspective. Academic Press, Elsevier; 2011.

[32] Rue H, Martino S, Lindgren F, Simpson D, Riebler A. INLA: Functions which allow to perform full Bayesian analysis of latent Gaussian models using Integrated Nested Laplace Approximaxion. 2014. Available: http://www.r-inla.org/

[33] Martino S, Rue H. Implementing Approximate Bayesian Inference using Integrated Nested Laplace Approximation: a manual for the inla program. Norway; 2010 Available: http://www.math.ntnu.no/~hrue/GMRFsim/manual.pdf

[34] Osenkowski JE, Paton PWC, Kraus D. Using Long-Term Constant-Effort Banding Data to Monitor Population Trends of Migratory Birds: A 33-Year Assessment of Adjacent Coastal Stations. Condor. 2012;114: 470–481.

[35] Schmidt JH, McIntyre CL, MacCluskie MC. Accounting for incomplete detection: What are we estimating and how might it affect long-term passerine monitoring programs? Biol Conserv. Elsevier Ltd; 2013;160: 130–139.

[36] Kéry M, Schmid H. Monitoring programs need to take into account imperfect species detectability. Basic Appl Ecol. 2004;5: 65–73.

[37] Bird Studies Canada, Environment Canada’s Canadian Wildlife Service, Ontario Nature, Ontario Field Ornithologists, Ontario Ministry of Natural Resources. Ontario Breeding Bird Atlas Website. 2006. Available: http://www.birdsontario.org/atlas/index.jsp

[38] Environment Canada. North American Breeding Bird Survey—Canadian Trends Website, Data—version 2012. 2012. Available: http://www.ec.gc.ca/ron-bbs/P005/A001/?lang=e&m=s&r=NOWA&p=L&t=26116

[39] Hussell DJT, Ralph CJ. Recommended methods for monitoring bird populations by counting and capture of migrants. Intensive Sites Tech Comm Migr Monit Counc. 1998; 1–18.

[40] Sanders CE, Mennill DJ. Acoustic monitoring of nocturnally migrating birds accurately assesses the timing and magnitude of migration through the Great Lakes. Condor. 2014;116: 371–383.

[41] Buler JJ, Dawson DK. Radar analysis of fall bird migration stopover sites in the northeastern U.S. Condor. 2014;116: 357–370.

[42] Taylor PD, Mackenzie SA, Thurber BG, Calvert AM, Mills AM, McGuire LP, et al Landscape movements of migratory birds and bats reveal an expanded scale of stopover. PLoS One. 2011;6: e27054 10.1371/journal.pone.0027054 22073253 PMC3207824

[43] Berthiaume É, Bélisle M, Savard J-P. Incorporating detectability into analyses of population trends based on hawk counts: A double-observer approach. Condor. 2009;111: 43–58.

[44] Bishop MA, Meyers PM, Furtsch McNeley P. A Method to estimate migrant shorebird numbers on the Copper River Delta, Alaska. J F Ornithol. 2000;71: 627–637.

[45] Bart J, Burnham KP, Dunn EH, Francis CM, Ralph CJ. Goals and strategies for estimating trends in landbird abundance. J Wildl Manage. 2004;68: 611–626.

[46] Harrison NM, Whitehouse MJ, Prince PA, Huin N. What problems do local habitat change represent for the Constant Effort Site ringing scheme? Ringing Migr. 2000;20: 1–8.

