# Supplementary material for: Modeling Systematic Change in Stopover Duration Does Not Improve Bias in Trends Estimated from Migration Counts
Source: PLoS One. 2015 Jun 18;10(6):e0130137. doi: 10.1371/journal.pone.0130137 (PMC4472725; doi:10.1371/journal.pone.0130137)
Supplement: S1 Appendix — (PDF) [file pone.0130137.s001.pdf]

S1 Appendix. R Code for migration count simulation.

```
# Function to simulate capture-recapture data under the JS Model in Kery and Schaub (2011) – Bayesian Population Analysis using WinBUGS.
# N Days does not vary among years
# Y1 = starting population size in year 1
# ndays = number of capture occasions (days in season)
# phi = survival - assumed constant in a year, but can vary among years
# p = probability of capture – assumed constant across days and years


simul.js <- function(nyears, Y1, trend, trend.err, nb.annual.size, ndays, mean.sigma2, sd.sigma2, sd.mu, c, a, phi.in, phi.in.1, phi.in.2, p.in, prob.move, xpred.err, sim, phi.type, out.dir) {

# set up NULL files for output

  out.data <- NULL # total pop size with stopover and detected population size

  years <- 1:nyears

## Log-linear population change:

  log.Yi <- log(Y1 * (1 + trend)^(0:(nyears - 1)))

## Add random year effects on log scale

  Yi <- exp(log.Yi + rnorm(nyears, trend, trend.err))

## add Negative Binomial noise around expected mean

  Yi <- rnbinom(n = nyears, size = nb.annual.size, mu = Yi)

# Define the parameter values for the daily distribution of counts

  for(i in 1:nyears) {

  n.occasions <- ndays
  date <- c(1:n.occasions)
  sigma2 <- rnorm(1, mean = mean.sigma2, sd = sd.sigma2)         # allows variation to vary among years
  mu <- rnorm(1, mean = n.occasions/2, sd = sd.mu)    # allows mean of distribution to vary among years

# NULL files for output
  s <- rep(NA, times = n.occasions)                               # probability of birds entering each day
  xpred <- rep(0, times = n.occasions)
  x <- rep(NA, times = n.occasions)
  b <- rep(NA, times = n.occasions)             # number individuals entering each day

# daily probability of entering, b, based on normal distribution:
  for(j in 1:n.occasions) {
        s[j] <- (2 * 3.14 * sigma2)^-0.5 * exp(-0.5*(j - mu)^2/sigma2)
        }
```

```
# modify s, so that on bad weather days, no birds migrate

  weather <- rbinom(n = n.occasions, size = 1, prob = prob.move)
  s <- s*weather/sum(s)

# daily number of birds entering, with autocorrelation

 for(j in 1:n.occasions){
		x[j] <- rnorm(1, xpred[j], xpred.err)
		xpred[j+1] <- a*x[j]
		b[j] <- rpois(1, lambda = Yi[i]*s[j]*exp(c*(x[j]-(j-1)/(n.occasions - 1) * xpred[n.occasions])))
		}
  B <- round(b*Yi[i]/sum(b), digits = 0); plot(B)
  Y <- sum(B)  # Annual total


# The following is based on Kery and Schaub 2011

  p <- rep(p.in, times = n.occasions)
  phi <- rep(phi.in[i], times = n.occasions) # so that survival can vary among years

  PHI <- matrix(rep(phi, (n.occasions-1)*Y), ncol = n.occasions-1,
		nrow = Y, byrow = T)
  P <- matrix(rep(p, n.occasions*Y), ncol=n.occasions, nrow = Y, byrow = T)

# NULL files
  CH.sur <- CH.p <- matrix(0, ncol = n.occasions, nrow = Y)
  CH.dur <- NULL

# define a vector with the occasion of entering the population
  ent.occ <- numeric()
  for(t in 1:n.occasions) {
		ent.occ <- c(ent.occ, rep(t, B[t]))
		}

# Simulate Arrival
  for(j in 1:length(ent.occ)) { #
  CH.sur[j, ent.occ[j]] <- 1 # write 1 when ind. enters the pop
		if(ent.occ[j] == n.occasions) next
		for(t in (ent.occ[j] + 1):n.occasions) {
		# Bernoulli trial: has individual survived occasion?
		sur <- rbinom(1, 1, PHI[j, t-1])
		ifelse(sur ==1, CH.sur[j, t] <- 1, break)
  } # t
  CH.dur[j] <- length(which(CH.sur[j,] == 1)) # number of days individual survived in reality (avail for
capture)
  } # j


# Simulate capture
```

```
  for(j in 1:Y) {
        CH.p[j,] <- rbinom(n.occasions, 1, P[j,])
        } #i

# Full capture-recapture matrix
  CH <- CH.sur * CH.p

# Remove individuals never captured

  cap.sum <- rowSums(CH)
  never <- which(cap.sum ==0)
  CH <- CH[-never,]

# Output "Actual" population size (new plus remaining individuals)
  tmp <- as.data.frame(colSums(CH.sur))
  names(tmp) <- "N.Avail"
  tmp$year <- i
  tmp$doy <- row.names(tmp)

# Output detected population size
  tmp2 <- as.data.frame(colSums(CH))
  names(tmp2) <- "count"
  tmp2$year <- i
  tmp2$doy <- row.names(tmp2)

# merge actual and detected population size
  tmp <- merge(tmp, tmp2, by = c("year", "doy"), all = TRUE)
  tmp$sim <- sim
  tmp$trend <- trend
  tmp$phi.type <- phi.type
  tmp$phi <- paste(phi.in.1, phi.in.2, sep = "")
  out.data <- rbind(out.data, tmp)


  } # end of year loop

  write.csv(out.data,
        file = paste(out.dir, "StopoverData.", trend.type, ".",
        phi.type, ".", phi.in.1, phi.in.2, ".", sim, ".NDETECTIONS.csv", sep = ""), row.names =
FALSE)

  } # end of function
```

# Code required to RUN simulation function

```
nsims <- 100
for(s in 1:nsims) {

simul.js(nyears = nyears, Y1 = 1000, trend = trend, trend.type, trend.err = 0.45, nb.annual.size = 5, ndays
= 65, mean.sigma2 = 50, sd.sigma2 = 10, sd.mu = 2.25, c = 1.6, a = 0.2, phi.in = phi.in, phi.in.1 =
phi.in.1, phi.in.2 = phi.in.2, p.in = 0.3, prob.move = 0.85, xpred.err = 1.25, sim = s, phi.type = phi.type,
out.dir = out.dir)
```

} # end of for loop
