# Supplementary material for: Modeling Systematic Change in Stopover Duration Does Not Improve Bias in Trends Estimated from Migration Counts
Source: PLoS One. 2015 Jun 18;10(6):e0130137. doi: 10.1371/journal.pone.0130137 (PMC4472725; doi:10.1371/journal.pone.0130137)
Supplement: S2 Appendix — (PDF) [file pone.0130137.s002.pdf]

S2 Appendix. R Code to run negative binomial model with first order auto-regressive random effects in INLA. Example R code to fit negative binomial model with an independent and identically distributed (IID) random effect for year, and a first order auto-regressive random effect (AR1) for day nested within year. Year and day effects were centered prior to analysis (cyear, cday)

```
require(INLA)
```

Set up model (can include or not include effect of phi). Alternative parameterization using -1

```
formula1 <- count ~ cyear - 1 + doy1 + doy2 + phi + f(fdoy,
   model = "ar1", replicate = year) + f(fyear, model = "iid")
```

Run model

```
inla.out <- inla(formula1, family = "nbinomial", data = in.data,
control.predictor = list(compute = TRUE), control.inla =
list(int.strategy = "grid"), control.compute = list(config =
TRUE))
```

Extract fitted values

```
in.data$pred1 <-  inla.out$summary.fitted.values[,"mean"]
```

Fixed effects

```
summary(inla.out)$fixed
```

Random effects

```
summary(inla.out)$hyperpar
```
