# Supplementary material for: Modeling Systematic Change in Stopover Duration Does Not Improve Bias in Trends Estimated from Migration Counts
Source: PLoS One. 2015 Jun 18;10(6):e0130137. doi: 10.1371/journal.pone.0130137 (PMC4472725; doi:10.1371/journal.pone.0130137)

S1 Fig. Simulated levels and pattern of change in daily probability of survival. Levels of constant, random, systematic or cyclic variation in daily survival probability (*phi*) tested for their influence on accuracy and precision of population trends derived from unmarked migration counts. Values shown for random variation in *phi* depict one draw from a random uniform distribution. For random, systematic and cyclic variation, *phi* varied between 0.2–0.7, 0.25–0.65, 0.3–0.6, 0.35–0.55, and 0.4–0.5.

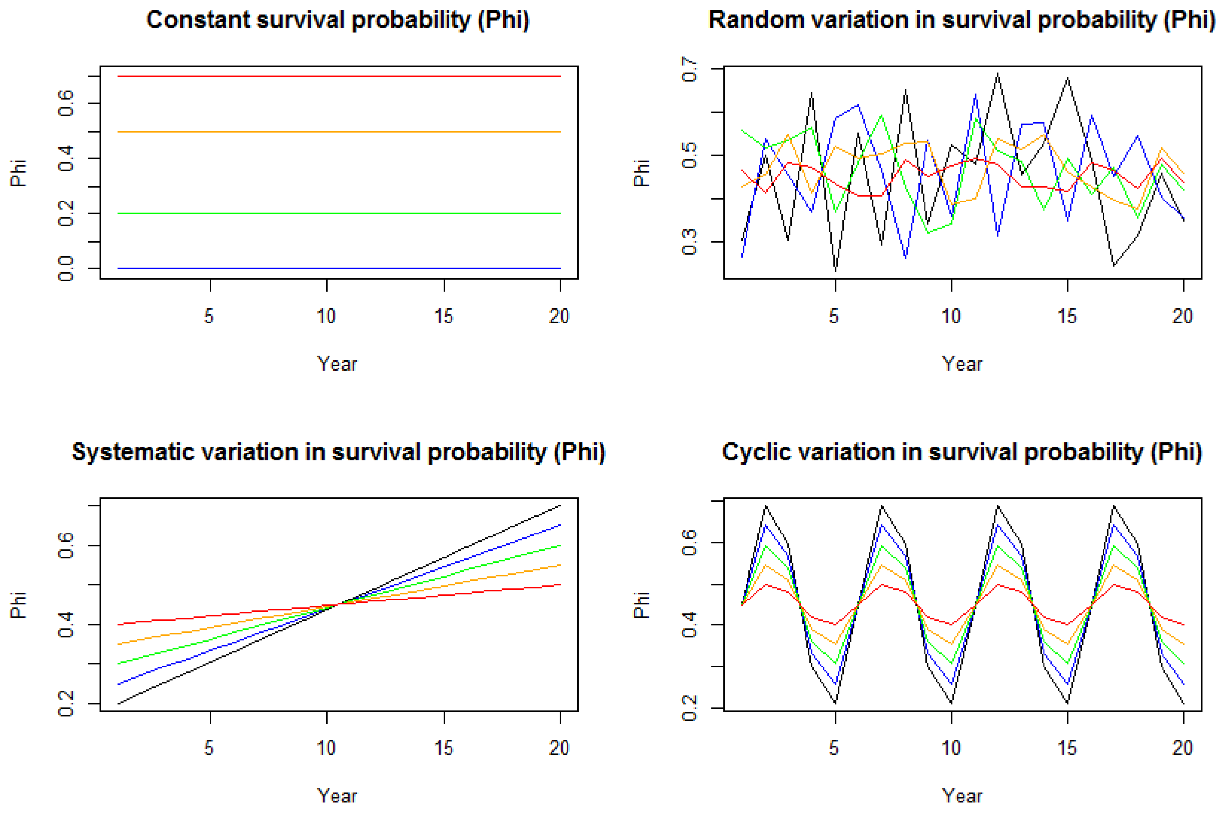

Supplement: S1 Fig — Levels of constant, random, systematic or cyclic variation in daily survival probability (phi), an index of stopover duration, tested for their influence on accuracy and precision of population trends derived from unmarked migration counts. Values shown for random variation in phi depict one draw from a random uniform distribution. For random, systematic and cyclic variation, phi varied between 0.2–0.7, 0.25–0.65, 0.3–0.6, 0.35–0.55, and 0.4–0.5. (PDF) [file pone.0130137.s003.pdf]
