# Supplementary material for: Modeling Systematic Change in Stopover Duration Does Not Improve Bias in Trends Estimated from Migration Counts
Source: PLoS One. 2015 Jun 18;10(6):e0130137. doi: 10.1371/journal.pone.0130137 (PMC4472725; doi:10.1371/journal.pone.0130137)

S2 Fig. Simulated increase in daily survival probability (1- probability of departure), and associated increase in stopover duration and detections per individual. Mean (SD) of stopover duration and number of sampling occasions during which an individual was detected across 100 datasets simulated to have a linear increase in daily survival probability from 0.2 to 0.7 over a 20-year period, constant probability of observer detection (0.3), and no underlying trend in population size (0%year^{-1}).

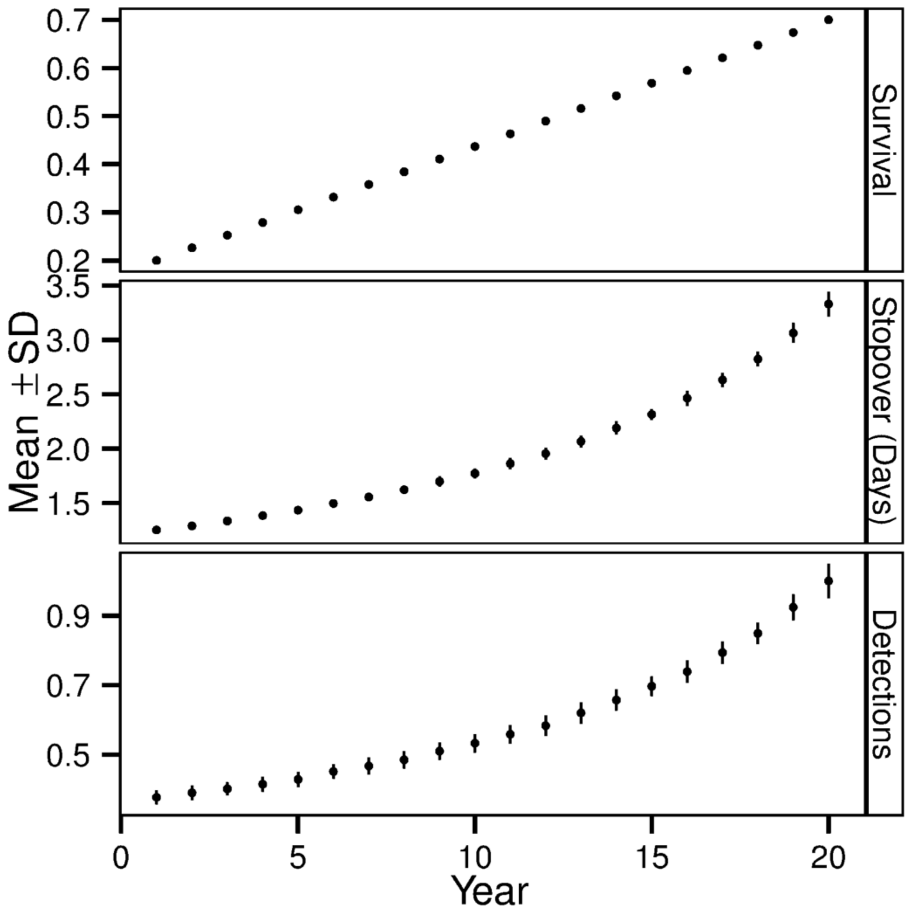

Supplement: S2 Fig — Mean (SD) of stopover duration and number of sampling occasions during which an individual was detected across 100 datasets simulated to have a linear increase in daily survival probability from 0.2 to 0.7 over a 20-year period, constant probability of observer detection (0.3), and no underlying trend in population size (0%year-1). (PDF) [file pone.0130137.s004.pdf]
