# Supplementary material for: Modeling Systematic Change in Stopover Duration Does Not Improve Bias in Trends Estimated from Migration Counts
Source: PLoS One. 2015 Jun 18;10(6):e0130137. doi: 10.1371/journal.pone.0130137 (PMC4472725; doi:10.1371/journal.pone.0130137)
Supplement: S1 Table — Values of simulation model parameters that varied among simulated factor levels, where: ‘trend’ specifies the rate of population trend simulated; ‘phi.type’ specifies whether daily probability of survival, and therefore stopover duration, was simulated to be constant, vary randomly or cyclically, or to increase linearly over time; ‘phi.in’ specifies the rate or range in daily survival probability simulated; ‘phi.in.1’ and ‘phi.in.2’ are the minimum and maximum values of daily survival, respectively; and ‘cycle.amp’ specifies the amplitude of cyclical change required to simulate the desired range in survival/stopover duration. See S1 Appendix for migration count simulation code in R. (PDF) [file pone.0130137.s005.pdf]

S1 Table. Migration count simulation parameterization. Values of simulation model parameters that varied among simulated datasets, where: 'trend' specifies the rate of population trend simulated (log scale); 'phi.type' specifies whether daily probability of survival was simulated to be constant, vary randomly or cyclically, or to increase linearly over time; 'phi.in' specifies the rate or range in survival probability simulated; 'phi.in.1' and 'phi.in.2' are the minimum and maximum values of survival, respectively; and 'cycle.amp' specifies the amplitude of cyclical change required to simulate the desired range in survival. See Appendix S1 for migration count simulation code in R.

trend	phi.type	phi.in	phi.in.1	phi.in.2	cycle.amp
-0.012 / 0/ 0.0096	constant	0	0	0	
		0.2	0.2	0.2	
		0.5	0.5	0.5	
		0.7	0.7	0.7	
-0.012 / 0/ 0.0096	random	0.2–0.7	0.2	0.7	
		0.25–0.65	0.25	0.65	
		0.3–0.6	0.3	0.6	
		0.35–0.55	0.35	0.55	
		0.4–0.5	0.4	0.5	
-0.012 / 0/ 0.0096	linear	0.2–0.7	0.2	0.7	
		0.25–0.65	0.25	0.65	
		0.3–0.6	0.3	0.6	
		0.35–0.55	0.35	0.55	
		0.4–0.5	0.4	0.5	
-0.012 / 0/ 0.0096	cyclic	0.2–0.7	0.2	0.7	0.25
		0.25–0.65	0.25	0.65	0.2
		0.3–0.6	0.3	0.6	0.15
		0.35–0.55	0.35	0.55	0.1
		0.4–0.5	0.4	0.5	0.05
