# Supplementary material for: Modeling Systematic Change in Stopover Duration Does Not Improve Bias in Trends Estimated from Migration Counts
Source: PLoS One. 2015 Jun 18;10(6):e0130137. doi: 10.1371/journal.pone.0130137 (PMC4472725; doi:10.1371/journal.pone.0130137)
Supplement: S2 Table — Mean, median, coefficient of variation (CV), minimum and maximum of migration counts collected daily at the tip station of the Long Point Bird Observatory, Ontario, Canada, during spring migration from 1961–2011. (PDF) [file pone.0130137.s006.pdf]

S2 Table. Summary of real white-throated sparrow (*Zonotrichia albicollis*) migration count data. Mean, median, coefficient of variation (CV), minimum and maximum of migration counts collected daily at the tip station of the Long Point Bird Observatory, Ontario, Canada, during spring migration from 1961–2011.

Variable	mean	median	CV	min	max
Annual Count	571	471	0.51	100	1160
Daily Count	15	4	1.88	0	141
Proportion 0-Observation Days	0.19	0.19	0.44	0	0.35
Observation Days/Season	44	43	0.18	31	70
