# Supplementary material for: Modeling Systematic Change in Stopover Duration Does Not Improve Bias in Trends Estimated from Migration Counts
Source: PLoS One. 2015 Jun 18;10(6):e0130137. doi: 10.1371/journal.pone.0130137 (PMC4472725; doi:10.1371/journal.pone.0130137)
Supplement: S6 Table — Mean, median and coefficient of variation (CV) of the number of observation days each season among 100 simulated datasets for each set of factor levels. Datasets were simulated to have either a declining population trend (-1.2%/year; “Decline”), no population change (0%/year; “NoChange”) or an increasing population trend (0.96%/year; “Increase”). Survival probability remained constant or varied randomly, cyclically or increased linearly over time. (PDF) [file pone.0130137.s010.pdf]

S6 Table. Summary of migration window length for simulated migration count data. Mean, median and coefficient of variation (CV) of the number of observation days each season among 100 simulated datasets for each set of factor levels. Datasets were simulated to have either a declining population trend (-1.2%/year; "Decline"), no population change (0%/year; "NoChange") or an increasing population trend (0.96%/year; "Increase"). Survival probability remained constant or varied randomly, cyclically or increased linearly over time.

		Mean			Median			CV		
Survival	Survival Probability	Decline	NoChange	Increase	Decline	NoChange	Increase	Decline	NoChange	Increase
Constant	0	35	36	36	36	37	37	0.18	0.18	0.17
	0.2	35	36	36	36	37	37	0.18	0.18	0.175
	0.5	35	36	37	36	37	38	0.18	0.17	0.17
	0.7	36	36	36	36	37	37	0.18	0.18	0.17
Random	0.20–0.70	39	40	40	40	40	41	0.17	0.16	0.16
	0.25–0.65	39	39	40	40	40	41	0.16	0.16	0.16
	0.30–0.60	39	39	40	40	40	41	0.16	0.16	0.155
	0.35–0.55	39	39	40	40	40	40	0.16	0.16	0.16
	0.40–0.50	39	39	40	39	40	41	0.16	0.16	0.16
Linear	0.20–0.70	39	40	40	40	41	41	0.17	0.16	0.17
	0.25–0.65	39	40	40	40	41	41	0.16	0.16	0.16
	0.30–0.60	39	39	40	40	40	40	0.16	0.16	0.16
	0.35–0.55	39	39	40	40	40	41	0.16	0.16	0.16
	0.40–0.50	38	39	40	39	40	41	0.16	0.16	0.15
Cyclic	0.20–0.70	39	40	40	40	41	41	0.17	0.17	0.17
	0.25–0.65	39	40	40	40	41	41	0.17	0.16	0.16
	0.30–0.60	39	39	40	40	40	41	0.17	0.16	0.16
	0.35–0.55	39	39	40	40	40	40	0.16	0.16	0.15
	0.40–0.50	39	39	39	40	40	40	0.16	0.16	0.16
