# Supplementary material for: Modeling Systematic Change in Stopover Duration Does Not Improve Bias in Trends Estimated from Migration Counts
Source: PLoS One. 2015 Jun 18;10(6):e0130137. doi: 10.1371/journal.pone.0130137 (PMC4472725; doi:10.1371/journal.pone.0130137)
Supplement: S7 Table — Mean (SD) of Pearson correlation coefficients of quantile-quantile (QQ) scores among 100 simulated migration count datasets with real white-throated sparrow (Zonotrichia albicollis) migration count data collected during spring migration at the tip station of the Long Point Bird Observatory in Ontario, Canada (1961–2011). A correlation coefficient of 1 suggests quantiles of each dataset originate from a similar distribution of counts. Four types of daily survival were tested: 1) constant (i.e., daily probability of survival was 0, 0.20, 0.50, or 0.70 across all years); or survival varied among years between 0.20–0.70, 0.25–0.65, 0.30–0.60, 0.35–0.55 and 0.40–0.50 either 2) randomly, 3) with linear/directional change, or 4) cyclically. A daily probability of survival of zero suggests all birds departed the count site within 24 hours (i.e., birds did not stop over at a site for extended periods with potential to be recaptured on subsequent counts). The lower correlation coefficients observed for simulations with constant phi is likely the result of lower mean daily and annual counts, a higher proportion of 0-observation days, and higher variability among daily counts compared to real data and to simulations with random, linear and cyclic variation in phi. (PDF) [file pone.0130137.s011.pdf]

S7 Table. Correlation of quantile-quantile plot scores comparing real and simulated migration count data. Mean (SD) of Pearson correlation coefficients of quantile-quantile (QQ) scores among 100 simulated migration count datasets with real white-throated sparrow (*Zonotrichia albicollis*) migration count data collected during spring migration at the tip station of the Long Point Bird Observatory in Ontario, Canada (1961–2011). A correlation coefficient of one suggests quantiles of each dataset originate from a similar distribution of counts. Four types of daily survival were tested: 1) constant (i.e., daily probability of survival was 0, 0.20, 0.50, or 0.70 across all years); or survival varied among years between 0.20 – 0.70, 0.25 – 0.65, 0.30 – 0.60, 0.35 – 0.55 and 0.40 – 0.50 either 2) randomly, 3) with linear/directional change, or 4) cyclically. A daily probability of survival of zero suggests all birds departed the count site within 24 hours (i.e., birds did not stop over at a site for extended periods with potential to be recaptured on subsequent counts). The lower correlation coefficients observed for simulations with constant phi is likely the result of lower mean daily and annual counts, a higher proportion of 0-observation days, and higher variability among daily counts compared to real data and to simulations with random, linear and cyclic variation in phi.

		Population Trend Type		
Survival	Survival Probability	Decline	No Change	Increase
Constant	0	0.92 (0.06)	0.92 (0.05)	0.93 (0.05)
	0.20	0.93 (0.04)	0.93 (0.04)	0.93 (0.05)
	0.50	0.93 (0.04)	0.93 (0.04)	0.94 (0.04)
	0.70	0.94 (0.04)	0.93 (0.05)	0.93 (0.05)
Random	0.20–0.70	0.98 (0.03)	0.97 (0.04)	0.97 (0.03)
	0.25–0.65	0.97 (0.03)	0.98 (0.02)	0.98 (0.03)
	0.30–0.60	0.98 (0.03)	0.97 (0.03)	0.98 (0.03)
	0.35–0.55	0.98 (0.02)	0.97 (0.03)	0.98 (0.03)
	0.40–0.50	0.98 (0.02)	0.98 (0.03)	0.97 (0.04)
Linear	0.20–0.70	0.98 (0.03)	0.98 (0.02)	0.98 (0.02)
	0.25–0.65	0.97 (0.03)	0.98 (0.03)	0.98 (0.03)
	0.30–0.60	0.97 (0.03)	0.97 (0.03)	0.98 (0.03)
	0.35–0.55	0.97 (0.03)	0.98 (0.03)	0.97 (0.04)
	0.40–0.50	0.98 (0.02)	0.98 (0.03)	0.97 (0.03)
Cyclic	0.20–0.70	0.98 (0.02)	0.98 (0.03)	0.98 (0.02)
	0.25–0.65	0.97 (0.03)	0.97 (0.03)	0.98 (0.03)
	0.30–0.60	0.98 (0.02)	0.98 (0.03)	0.97 (0.03)
	0.35–0.55	0.98 (0.02)	0.97 (0.03)	0.97 (0.03)
	0.40–0.50	0.98 (0.03)	0.97 (0.03)	0.97 (0.04)
